# Minimally Invasive Cell-Free Human Embryo Aneuploidy Testing (miPGT-A) Utilizing Combined Spent Embryo Culture Medium and Blastocoel Fluid –Towards Development of a Clinical Assay

**DOI:** 10.1038/s41598-020-64335-3

**Published:** 2020-04-29

**Authors:** Valeriy Kuznyetsov, Svetlana Madjunkova, Rina Abramov, Ran Antes, Zenon Ibarrientos, Gelareh Motamedi, Afsaneh Zaman, Iryna Kuznyetsova, Clifford L. Librach

**Affiliations:** 1 CReATe Fertility Centre, Toronto, Canada; 2 Department of Obstetrics and Gynecology, University of Toronto, Toronto, ON Canada; 3 Department of Physiology and Institute of Medical Sciences, University of Toronto, Toronto, ON Canada; 4 Department of Gynecology, Women’s College Hospital, Toronto, ON Canada

**Keywords:** Developmental biology, Genetics, Endocrinology

## Abstract

Preimplantation genetic testing for aneuploidies (PGT-A) using trophectoderm (TE) biopsy samples is labour intensive, invasive, and subject to sampling bias. In this study, we report on the efficacy and factors affecting accuracy of a technique we pioneered for minimally invasive preimplantation genetic testing for aneuploidy (miPGT-A). Our technique uses cell-free embryonic DNA (cfeDNA) in spent embryo culture medium (SEM) combined with blastocoel fluid (BF) to increase the amount of assayable cfeDNA. We compared miPGT-A results (n = 145 embryos) with standard PGT-A analysis of the corresponding trophectoderm biopsy. We found that accuracy of miPGT was not related to blastocyst morphological grade. The overall concordance rate per sample for euploidy/aneuploidy status between miPGT-A and TE biopsy samples was 88/90 (97.8%), and was not different between good 47/48 (97.9%) and moderate/low quality blastocysts 41/42 (97.9%) (p > 0.05). Importantly, we also discovered that for cfeDNA analysis, the SurePlex whole genome amplification (WGA) kit can be utilized without an additional cell lysis/extraction DNA step; this efficiency likely reduces the risk of maternal contamination. Regarding origin of embryonic cfeDNA, the average amount of miPGT-A WGA-DNA we obtained from blastocysts with different morphological grades, as well as the size miPGT-A WGA-DNA fragments, suggest that it is unlikely that apoptosis and necrosis are only mechanisms of DNA release from the inner cell mass (ICM) and TE into BF and SEM.

## Introduction

Preimplantation genetic testing for aneuploidies (PGT-A) using trophectoderm (TE) biopsy and next generation sequencing (NGS) as a testing platform for embryo selection has significantly improved ongoing pregnancy rates per transfer shortening the time to pregnancy in addition to the reduction of multiple pregnancies by transferring single euploid embryos, reduction in miscarriage rates and reduced risk of aneuploid pregnancies^1–4^. However, there are three main challenges of the preimplantation genetic testing associated with trophectoderm biopsy samples: (1) TE biopsy is labour intensive^5–7^; (2) TE biopsy is invasive^8,9^; and (3) it is subject to sampling bias - TE biopsy may not accurately represent the inner cell mass (ICM) and remainder of the TE^10–12^. Furthermore, although there has been no reported increase in the risk of adverse perinatal outcomes, such as pre-term birth and low birth weight, following invasive PGT compared with IVF without embryo biopsy, conclusive evidence regarding the long-term health of the offspring after embryo biopsy will take some time to obtain^5,13,14^.

Recently, cell-free embryonic nuclear DNA (cfeDNA) has been found in both blastocoel fluid^15–17^ and spent embryo culture medium^18–20^. Non-invasive preimplantation genetic testing (NIPGT) or minimally invasive preimplantation genetic testing (miPGT) using the cfeDNA of spent embryo culture medium (SEM) and/or blastocoel fluid (BF) has the potential to eliminate the need for embryo biopsy, thereby avoiding potential risks related to that invasive procedure^21–23^. Moreover, NIPGT/miPGT is less labour intensive and potentially more cost-effective method. In addition, NIPGT-A, which is based on sequencing of cfeDNA likely released from both TE and ICMcells^24,25^, may better represent the entire embryo compared to TE biopsy alone^26–28^.

Currently, there are three major ongoing research approaches regarding how to collect cell-free embryonic DNA for non-invasive or minimally invasive aneuploidy testing^29^:

1. Blastocoel fluid aspiration using an ICSI pipette^5,16,30^.
2. Spent embryo culture medium collection^22,31–34^.
3. Combined spent embryo culture medium and blastocoel fluid (SEM + BF) collection without blastocoel fluid aspiration^25,28,32,35^.

Attempts to use cfeDNA for non-invasive preimplantation aneuploidy testing bring to light several factors that could potentially affect the accuracy of this approach reducing concordance rates with TE biopsy results. These include maternal contamination by cumulus and corona cells^33^, cfeDNA degradation, low amounts of cfeDNA, variable DNA amplification efficacy and yield^7,36,37^, and short DNA fragments^17^.

Previous studies from our research group as well as others, have shown that cfeDNA testing using spent embryo culture medium on days 5 or 6 has the potential to detect chromosomal aneuploidy^22,25,31,33,35,38^ and monogenic disorders^5,19,39^. In addition, NIPGT-A, based on sequencing of cfeDNA likely released from both TE and ICM cells, may better represent the entire embryo compared to TE biopsy alone^30,37–39^. The mechanism(s) underlying the release of embryonic DNA in spent embryo culture medium and blastocoel fluid remain unclear^7^. Several authors have hypothesized that cfeDNA is correlated with apoptotic events^7,26,40^. If this is true, it should follow that lower quality blastocysts, which generally have higher degrees of apoptosis, would result in a higher quantity of cfeDNA release and thus more accurate results from aneuploidy testing.

High concordance rates between NIPGT-A and TE biopsy^23,24,29,39^, and between NIPGT-A and whole blastocysts^23,24,29^ were reported by several groups. However, there are several very important issues still need to be addressed before routine clinical application of NIPGT. These include: minimization of maternal DNA contamination risk, determining thefactors affectingaccuracy, and optimization of the WGA protocol for cfeDNA.

In this report, we have assessed the accuracy and reliability of utilizing cfeDNA in SEM + BF samples for blastocyst chromosomal status detection in comparison to corresponding TE biopsy samples in a larger cohort of fresh cultured embryos than our previous publication^25^. We have also evaluated several important factors that could influence this method, including: (1) quantity of amplified cfeDNA obtained; (2) the effect of blastocyst morphological grades on cfeDNA; (3) average size of WGA-DNA fragments from good and moderate/low quality blastocysts, and (4) whole genome amplification of cfeDNA from SEM + BF samples with or without a cell lysis/extraction enzymatic step.

## Results

### Blastocyst morphology had no effect on cfeDNA quantity and the mean size of WGA-DNA fragments miPGT samples

Table 1 shows analysis of the amount of amplified DNA and fragment sizes from each of the samples. The amount was highest in TE biopsy samples. Blank medium negative control samples associated with each sample that underwent WGA showed no amplification in all cases. Both the average size of WGA-DNA fragments and amount of amplified DNA from miPGT samples from good quality blastocysts (≥BB) (n = 55) vs. moderate/low quality blastocysts (<BB) (n = 47) was not statistically different (Table 1).However, the amount of amplified DNA and average size of WGA-DNA fragments from TE biopsy samples were significantly different from amplified miPGT samples from both good quality and moderate/low quality blastocysts.

**Table 1:** Blastocyst quality, and amount and size of amplified nuclear DNA in miPGT samples.

Types of samples	Amplification rate (%)	WGA-DNA amount range (ng/µl)	Average WGA-DNA amount (ng/µl)	Average size of WGA-DNA fragments	Informative NGS results (%)
miPGT, ≥BB	55/55 (100)	5.1 to 30.0	14.6 ± 4.9*	735.6 ± 27.1 bp*	48/55 (87.3)*
miPGT, <BB	47/47 (100)	6.3 to 36.0	15.9 ± 6.0*	746.9 ± 42.6 bp*	42/47 (89.4)*
miPGT, total	102/102 (100)	5.1 to 36.0	15.2 ± 5.4*	739.5 ± 22.7*	90/102 (88.2)*
TE biopsy	102/102 (100)	25.0 to 46.0	31.3 ± 1.2**	820.0 ± 32.5 bp**	100/102 (98.0)**

*Not statistically significant difference between the values in the same column.

**Statistically significant difference between the values in the same column.

### Blastocyst morphology had no effect on the rate of miPGT-A informative results or concordance compared to standard PGT-A

Informative NGS results (Table 2) were obtained for 98.0% of TE biopsies and for 88.2% of miPGT samples (87.3% for good quality and 89.4% for moderate/low quality blastocysts; p > 0.05). The overall concordance rate per sample for whole chromosome copy number abnormalities for euploidy/aneuploidy status between miPGT and TE biopsy samples was 88/90 (97.8%), and was not different between good 47/48 (97.9%) and moderate/low quality blastocysts 41/42 (97.9%) (p > 0.05) (Tables 2 and 3). miPGT-A analysis correlated with PGT-A results for gender (100%) and aneuploidy in 92.6% miPGT samples. Aneuploidy/euploidy concordance rate did not depend on blastocyst quality (Table 2).

**Table 2:** Blastocyst quality and concordance rate for whole chromosome copy number abnormalities between miPGT samples and corresponding TE biopsy samples.

Type of samples	Ploidy status (%)		Gender (%)	Euploid samples (%)	Aneuploid samples (%)
	Per sample	Per chromosome			
miPGT, ≥BB vs. TE	47/48 (97.9)*	1128/1152 (97.9)*	48/48 (100)	37/38 (97.4)*	10/11 (90.9)*
miPGT, <BB vs. TE	41/42 (97.6)*	984/1008 (97.6)*	42/42 (100)	26/26 (100)*	15/16 (93.8)*
miPGT, total vs. TE	88/90 (97.8)	2112/2160 (97.8)	90/90 (100)	63/64 (98.4)	25/27 (92.6)

Ploidy status - euploid or aneuploidy.

Aneuploid – whole/segmental chromosome aneuploidy.

*Not statistically significant difference between the values in the same column.

**Table 3:** Summary of NGS results from all trophectoderm biopsy and miPGT samples obtained from the corresponding blastocyst.

Embryo number	TE biopsy	miPGT, ≥BB
***Euploid-euploid***		
1	XX; normal	XX; normal
2	XX; normal	XX; normal
3	XX; normal	XX; normal
4	XX; normal	XX; normal
5	XX; normal	XX; normal
6	XX; normal	XX; normal
7	XX; normal	XX; normal
8	XY; normal	XY; normal
9	XX; normal	XX; normal
10	XX; normal	XX; normal
11	XY; normal	XY; normal
12	XY; normal	XY; normal
13	XX; normal	XX; normal
14	XY; normal	XY; normal
15	XY; normal	XY; normal
16	XY; normal	XY; normal
17	XY; normal	XY; normal
18	XX; normal	XX; normal
19	XY; normal	XY; normal
20	XY; normal	XY; normal
21	XX; normal	XX; normal
22	XY; normal	XY; normal
23	XY; normal	XY; normal
24	XX; normal	XX. normal
25	XY; normal	XY; normal
26	XX; normal	XX; normal
***Euploid-mosaic***		
27	XY; normal	XY; mosaic −8 (70%)
28	XY; mosaic −9 (30%)	XY; normal
29	XX; mosaic −15 (20%)	XX; normal
30	XX; mosaic: −8p (35%), −9p (35%)	XX; normal
31	XX; normal	XX; mosaic −9 (70%)
***Mosaic-mosaic***		
32	XY; mosaic −4q (118.6 Mb, 20%)	XY; mosaic +13q21.1 −q 31.3 (34.84 Mb, 60%)
33	XY; mosaic loss: (−4q22.1-q35.2, 100 Mb, 20%)*	XY; mosaic gain: (+4q 22.1-q35.2, 100 Mb, 30%)*
34	XY; mosaic: +8q (50%), −5q15-q35.3 (88MB, 35%)	XY; mosaic −3 (70%)
35	XY; mosaic −16 (60%)	XY; mosaic −17 (−70%)
36	XX; mosaic: +8 (50%), +19 (50%)	XX; mosaic: −17 (30%)
37	XX; mosaic: −22 (60%)	XX; mosaic: −22 (70%)
***Segmental aneuploid-euploid***		
38	XX; normal	XX; −5q23.3-q35.3 (51.71 Mb)
***Aneuploid-aneuploid***		
39	XX; −14	XX; −14
40	XX; −15	XX; −15
41	XY; +16, +21	XY; +16, +21
42	XX; +16	XX; +16
43	XY; −7, −15, −18	XY; −7, −15, −18
44	XX; −22	XX; −22
45	XX; −13	XX; −13
46	XY; +16	XY; +16
47	XY; −7	XY; −7
48	XY; +21	XY; +21
Embryo number	TE biopsy	miPGT, <BB
***Euploid-euploid***		
1	XX; normal	XX; normal
2	XY; normal	XY; normal
3	XY; normal	XY; normal
4	XX; normal	XX; normal
5	XY; normal	XY; normal
6	XX; normal	XX; normal
7	XX; normal	XX; normal
8	XX; normal	XX; normal
9	XY; normal	XY; normal
10	XX; normal	XX; normal
11	XX; normal	XX; normal
12	XY; normal	XY; normal
13	XY; normal	XY; normal
14	XX; normal	XX; normal
15	XY; normal	XY; normal
16	XX; normal	XX; normal
17	XX; normal	XX; normal
***Euploid-mosaic***		
18	XX; mosaic loss: (−3p.26.3 ± p25.2, 12 Mb, 35%)	XX; normal
19	XX; mosaic gain: +12p13.33-q23.3 (105.5 Mb, 45%)	XX; normal
20	XY; mosaic +16p (40%)	XY; normal
21	XX; mosaic −8 (40%)	XX; normal
***Mosaic-mosaic***		
22	XY; mosaic +6q22.1-q.25.2 (38.5 Mb, 45%), mosaic −15 (50%)	XY; mosaic −8 (50%)
23	XY; mosaic loss: −3q26.31-q29 (24.4 Mb, 65%)	XY; mosaic loss: −3q26.31-q29 (24.4 Mb, 65%)
24	XX; mosaic: +4 (30%), +5 (30%), +8 (30%), +10 (50%)	XX; mosaic: +5 (40%), +10 (40%)
25	XY; mosaic + 14q11.2-q32.33, 84.69 Mb, 65%*	XY; mosaic −14q11.2-q32.33, 84.69 Mb, 60%*
26	XY; mosaic +4 (40%)	XY; mosaic +4 (50%)
***Complex Aneuploid-mosaic***		
27	XX; −18	XX; mosaic −18q12.2-q23 (43.75 Mb, 40%)
***Aneuploid-aneuploid***		
28	XY; −5, −13	XY; −5, −13
29	XX; +19	XX; +1919
30	XY; +10, −11, −20	XY; +10, −11, −20
31	XY; +11, −16, +22	XY; +9, +11, −16, +22
32	XX; +22	XX; +22
33	XX; +13, +19, −21	XX; +13, +19, −21
34	XY; +11, mosaic gain: +10q23.31-26.3 (44.26MB, 50%)	XY; +10, +11, +16
35	XX; −3p26.3-p22.1 (39, 8 Mb)	XX; −3p26.3-p22.1 (39, 8 Mb)
36	XY; −17, +21, mosaic: +1p31.1-p21.1 (39 Mb, 30%)	XY; −17, +21
37	XY; −19	XY; −19
38	XY; −4, −8, +9, +18	XY; −4, −8, +9, +18
39	XY; +22	XY; +22
40	XX; −11	XX; −11
41	XY; −22	XY; −22
42	XY; +16	XY; +16

*Mosaic-complementary in terms of chromosomal gain versus loss between TE biopsy and miPGT samples.

Blastocysts were grouped based on their static morphology in good if graded as ≥1/2 BB, or moderate/low if graded <1/2BB.

**Figure 1:**
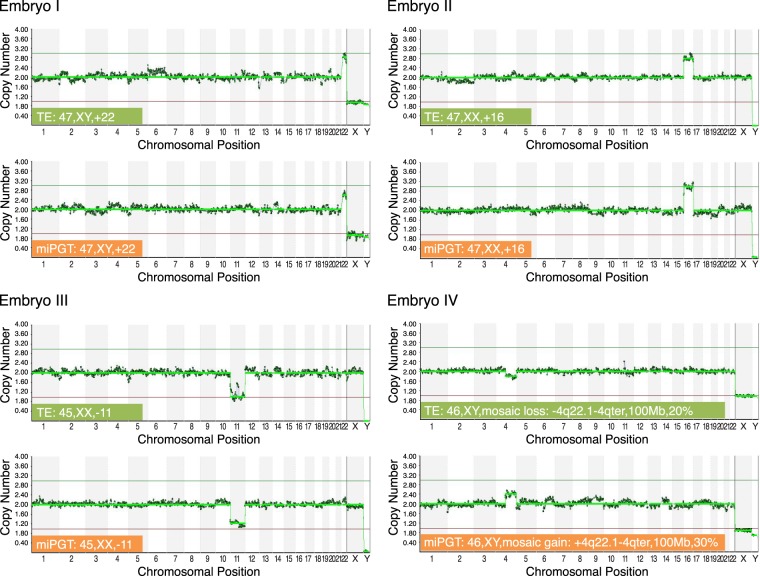
Four examples of NGS results representing 24 chromosome copy number plots from TE biopsy and corresponding miPGT samples with concordant results for aneuploidy in three examples and mosaic-complementary in terms of loss versus gain on chromosome 4q (segmental chromosomal mosaicism) in the fourth embryo.

A detailed summary of miPGT-A and PGT-A results for paired samples of embryos with good and moderate/poor morphology are presented in Table 3. Figure 1 presents CNV plots from representative samples from this group.

Mosaicism results were complex when comparing PGT-A with miPGT-A (Table 3). There were 4 cases in which there was relatively full concordance between PGT-A with miPGT-Ain terms of mosaicism and the chromosome involved. In one case (embryo #33, Table 3), there was a mosaic segmental loss on chromosome 4q in the TE sample versus a complementary gain on chromosome 4q in the miPGT sample. There were 4 other cases that showed mosaicism in both PGT-A and miPGT-A samples but there was discordance as to which chromosome was involved. Interestingly, there were 3 cases in which miPGT-A showed euploidy and the PGT-A showed mosaicism, and 2 cases in which the PGT-A showed euploidy and the miPGT-A showed mosaicism (Table 3). We found no obvious differences in the rate of mosaicism detected or discordance rate between PGT-A and miPGT-A for good quality vs medium/low quality embryos, but the number of mosaic cases in these two cohorts was too small to make any accurate conclusions regarding this comparison (Table 3 and Supplement Table S1).

### DNA amplification rate, amount of amplified cfeDNA and NGS results from miPGT samples with or without cell lysis/extraction enzyme step

The second objective of this study was to determine the accuracy, efficacy and reliability of whole genome amplification (WGA) to determine ploidy status of the blastocyst using combined SEM + BF samples with or without using the cell lysis/extraction enzyme step before WGA on separate aliquots from the same pool of SEM + BF collected for miPGT-A analysis.

We collected 2 aliquots from SEM + BF (miPGT samples) (n = 86) from 43 additional blastocysts. We analysed the amount of amplified DNA and NGS data of the 86 miPGT samples and corresponding 43 trophectoderm biopsy samples obtained from fresh blastocysts that underwent PGT-A cycles from. The first aliquot (miPGT-1) SEM + BF sample followed the standard SurePlex WGA protocol which starts with 5ul of sample and a cell lysis step. The second aliquot from the same SEM + BF pool (miPGT-2) was amplified following a modified WGA SurePlex protocol that starts with 10ul of sample and the direct pre-amplification. WGA products of miPGT-1 and miPGT-2 samples were compared with each other, and results were compared with the corresponding trophectoderm biopsy sample used as a control.

The amount of concentrated amplified cfeDNA from miPGT-1 samples was higher than in miPGT-2 samples, however this was not statistically significant. Respective blank medium (negative control) associated with each sample showed no amplification in all cases (Table 4).

**Table 4:** Amount of concentrated amplified nuclear DNA in miPGT-1 (WGA with cell lysis) and miPGT-2 (WGA without cell lysis) samples.

Types of samples	Amplification rate (%)	WGA-DNA range (ng/µl)	WGA-DNA concentration (ng/µl)	Informative NGS results (%)
miPGT-1 (with cell lysis)	43/43 (100)	6.3 to 85.9	37.3 ± 19.4*	40/43 (93.0)*
miPGT-2 (without cell lysis)	43/43 (100)	10.2 to 72.7	32.8 ± 16.3*	38/43 (88.4)*
miPGT, total	86/86 (100)	6.3 to 85.9	35.05 ± 17.85	78/86 (90.7)
TE biopsy**	43/43 (100)	32.3 to 52.4	43.2 ± 3.5	41/43 (95.3)*

*Not statistically significant Difference between the values in the same column.

**Not concentrated amplified DNA in TE biopsy sample.

**Figure 2:**
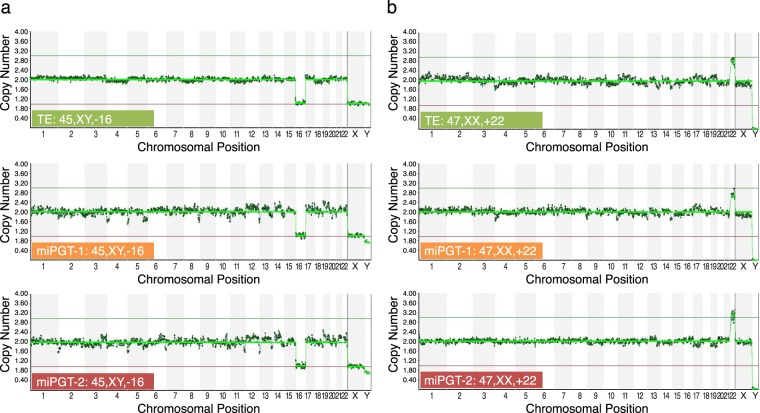
Example of NGS results from TE biopsy and corresponding miPGT-1 (WGA with cell lysis) and miPGT-2 (WGA without cell lysis) samples.

Informative NGS results (Table 5) were obtained for 95.3% trophectoderm biopsies, for 93.0% of miPGT-1, and for 88.4% of miPGT-2 samples; the difference was not statistically significant. There was a high concordance rate (Table 5 and Supplementary Table S1) per sample for whole chromosome copy number abnormalities between: 1) miPGT-1 and TE biopsy samples (97.4%), 2) miPGT-2 and TE biopsy samples (97.2%) and 3) miPGT-1 and miPGT-2 samples (100%). miPGT correctly determined the gender of the embryos and aneuploidy for all chromosomes in minimally invasive samples (Supplementary Table S1, Fig. 2). Aneuploid embryo #43 (Supplementary Table S1) had complimentary aneuploidy, in term of gain versus loss of chromosome 9, between TE biopsy and both miPGT samples. Interestingly, analysis of the aneuploid embryo #40 (Supplementary Table S1) showed that the TE sample was trisomy 13, while both miPGT-1 and miPGT-2 had monosomy 20. We were unable to test the inner cell mass and rest of the TE for these embryos because we did not have patient consent for donation of the embryo to research.

**Table 5:** Concordance rate for whole chromosome copy number abnormalities between corresponding miPGT-1(WGA with cell lysis), miPGT-2 (WGA without cell lysis) and TE samples.

Type of samples	Ploidy status (%)		Gender (%)	Euploid embryos (%)	Aneuploid embryos (%)
	Per sample	Per chromosome			
miPGT-1 vs. TE	37/38 (97.4)*	888/912 (97.4)*	38/38 (100)	23/24 (95.8)*	14/15 (93.3)*
miPGT-2 vs. TE	35/36 (97.2)*	840/864 (97.2)*	36/36 (100)	22/23 (95.7)*	13/14 (92.9)*
miPGT-1 vs. miPGT-2	38/38 (100)*	912/912 (100)*	38/38 (100)	24/24 (100)*	14/14 (100)*

Ploidy status – euploid or aneuploidy.

Aneuploid – whole/segmental chromosome aneuploidy.

*Not statistically significant difference between the values in the same column.

## Discussion

It has been recently reported that only ∼8% of DNA in spent embryo culture medium is embryonic in origin^33,40^.This could potentially impact the analysis of embryonic DNA from spent culture media. To minimize maternal contamination in our studies, we modified the procedure steps during Day 0 to Day 4 of embryo culture to include careful removal of residual corona cells by pipetting and washing^25^. Using a fluorescently labelled short tandem repeat (STR) marker for the analysis of both embryonic DNA (TE cells) and miPGT samples, we were able to confirm that this step minimizes maternal contamination. This is critical to avoid misdiagnosis with NIPGT-A.

In addition, our approach to transfer embryos into individual fresh droplets of medium on Day 4 and using SEM + BF samples obtained after Day 5/6 culture^25^, promotes a yield of less degraded cfeDNA. This method considers the embryonic genome activation stage in human embryos^41,42^ and the number of blastomeres on Day 4 versus Day 3. Our approach was also adopted in two recent studies^24,34^ that showed the superiority of using spent embryo fresh culture media from embryos cultured from day 4 today 5/6 for non-invasive PGT-A, compared to samples collected from a more extended culture period, which likely results in a more degraded cfeDNA sample.

The issue of whether nucleic acids can penetrate though the zona pellucida is still an unsolved question. We hypothesized that assisted hatching (AH) may facilitate the release of a high molecular weight cfeDNA from BF into the culture medium. In our studies AH was performed on Day 4. For better passage of embryonic DNA, some researchers have tried to use zona opening on Day 3^22,33^. In other studies assisted hatching was not performed prior to conducting the TE biopsy^34^. Ho *et al*. found that assisted hatching on Day 3 did not influence cfeDNA concentration or accuracy of cfeDNA sequencing for aneuploidy screening^31^. CfeDNA isolated from spent embryo culture medium on Day 2/3 has a low molecular weight, where cellular fragmentation may also play a role^20^. Vera-Rodriguez *et al*. pointed out that samples isolated from spent embryo culture medium could contain high molecular weight DNA or sheared DNA^33^. We have not measured the amount of cfeDNA in D4-D5/6 embryo culture media, with or without using laser zona opening on Day 4 to test this hypothesis.

The mechanism(s) underlying the release of embryonic DNA into the culture medium remain unclear and the origin of these DNA fragments is unknown^2,7,43^. The most popular hypothesis is that nucleosome sized DNA fragments (~180–200 bp) are being released from cells as a result of apoptosis^27,38,40,44^. Another mechanism that could contribute cell free DNA with longer sized DNA fragments is necrosis^40^.

Previous studies have reported that the concentration of cfeDNA correlates with apoptotic events^44^. We hypothesized that lower grade blastocysts, which tend to have a higher rate of apoptosis, will release a higher quantity of cfeDNA into the medium. Recently Ho *et al*. found that blastocyst morphology did not influence cfeDNA concentration in spent embryo culture medium or accuracy for aneuploidy screening^31^. Rule K *et al*., indicated that cfeDNA in blastocoel fluid positively correlates with a high embryonic morphology score, which suggests that the better the embryo morphology, the higher the cfeDNA concentration^44^.

The results of our study, which represents the largest number of blastocysts tested using spent embryo culture medium combined with blastocoel fluid, demonstrated that the morphological grade of blastocysts does not affect the rate of informative NGS results from cfeDNA. The amount of amplified DNA from good quality blastocysts was slightly lower than that from moderate/low quality blastocysts, however the difference was not statistically significant. The concordance rate per sample for whole chromosome copy number between miPGT and TE biopsy samples (Tables 2 and 3), for both good and moderate/low quality blastocysts, was not statistically different. The mean size of WGA-DNA fragments derived from miPGT samples from good quality blastocysts and from moderate/low quality blastocysts was not statistically different (Table 1). Considering the amount of miPGT-A WGA-DNA from different blastocysts and the size of miPGT-A WGA-DNA fragments (not close to nucleosomal size), cell apoptosis may not be the only mechanism for DNA release from the ICM and TE into BF and SEM. Therefore, other mechanisms for release of embryonic DNA are probably involved. One possibility is that embryonic DNA in culture media may derive from cells damaged due to the laser pulses used during artificial shrinkage, although, as mentioned by us and Jiao *et al*.^32^, the single laser pulse was used at the junction of TE cells located far away from the inner cell mass. Extrachromosomal microDNAs^7^could also be a source of cfeDNA in spent culture medium. Production of these microDNAs is a part of normal cellular physiology and has been linked to transcriptional activity and mismatch repair. Extrachromosomal microDNAs vary in size from 60 to 2000 base pairs. They are abundant in all tissue types of mammalian cells, including sperm^41^. In contrast to accumulation of embryonic DNA in culture medium by apoptosis or necrosis, this mechanism would not necessarily depend on cell death^7^. Functional aspect of the DNA or RNA released by the developing preimplantation embryo is unknown and whether it is involved in cellular communication is still a subject of research. Extracellular vesicles (EVs) in blastocoel fluid and embryo culture medium as a transport vehicle may contain packed DNA to transmit information between the cells of trophectoderm and inner cell mass^40^. Similar cross-talk by means of EVs transferring miRNAs and other molecules (mRNAs, DNA, lipids and proteins) has been described among cells^43,45^. We have also recently reported that EVs appear to be able to traverse the zona pellucida when human embryos are cultured *in vitro*, which supports this hypothesis^46^.

Since cell-free embryonic DNA consists of relatively short DNA fragments, the analysis of spent culture medium requires modifications of the standard WGA protocol. We hypothesized that for cfeDNA analysis, the SurePlex whole genome amplification kit can be used without the need for a cell lysis/extraction DNA step. Here we report that miPGT-1 and miPGT-2 samples (Table 5 and Supplement Table S1) show a similar high concordance rate with corresponding TE biopsy samples for a chromosome copy number. In our opinion, amplification of cfeDNA without using the cell lysis/extraction DNA step has the potential to reduce the risk of maternal contamination of NIPGT/miPGT samples by residual cumulus/corona cells.

Assisted hatching is generally performed using a laser pulse prior to blastocyst vitrification, resulting in artificial shrinkage of the blastocoel. This helps to prevent injury from intracellular ice formation and has been shown to improve clinical outcomes^47^. A single laser pulse creates an opening in the zona pellucida at the cellular junction of trophectoderm cells located far away from the inner cell mass^30,48,49^. Therefore, our approach to use laser zona opening on Day 4 together with the laser (or microneedle) collapsing of blastocysts prior to TE biopsy for cfeDNA collection should have no negative impact on blastocyst development and does not require an additional laser (or microneedle) collapsing step before blastocyst vitrification as in current clinical practice^49^.

We have previously shown that collection of both spent embryo culture media and blastocoel fluid as one non-invasive sample increases the quantity and quality of cfeDNA for aneuploidy testing, compared with either spent embryo culture media only, or blastocoel fluid only^25^. In our study, some WGA-miPGT DNA samples had chaotic or inconclusive results due to low or poor quality cfeDNA that led to noisy NGS profiles. The same issue was noted by Rubio *et al*.^34^. Noisy DNA profiles could also be attributed to maternal contamination by residual cumulus or corona cells. Therefore, if used in parallel with TE biopsy, NIPGT-A/miPGT may improve testing efficacy and accuracy by acting as a backup source of embryonic DNA in cases of inconclusive TE biopsy results; this would also obviate the need for re-biopsy.

To our knowledge, only few studies have been conducted comparing NIPGT-A/miPGT samples to corresponding TE biopsy samples and the whole blastocyst as a gold standard control. Results from our previous study^25^, as well as Li *et al*.^35^ and Huang *et al*.^24^, revealed that concordance rates for both embryo ploidy and chromosome copy number between NIPGT-A samples and whole blastocyst were higher than between TE biopsy and whole blastocyst. Conversely, the study of Ho *et al*.^31^ showed that the concordance rate for embryo ploidy between NIPGT-A samples and whole blastocyst was lower than between TE biopsy and whole blastocyst. In contrast, Jiao *et al*.^32^ reported similar concordance between NIPGT-A and TE biopsy samples and between NIGPT-A and the whole blastocyst. Considering results obtained for the non-invasive samples, Huang *et al*.^24^ also suggested that NIPGT-A is less prone to errors associated with embryo mosaicism and is more reliable than TE biopsy PGT-A.

An embryo transfer performed on November 2017 of a euploid blastocyst tested by both TE biopsy and cfeDNA from a combined SEM + BF miPGT-A sample at the CReATe Fertility Centre, Toronto, resulted in a healthy boy, born at full term. To our knowledge, this is the first report where two sources of embryonic DNA (SEM + BF and corresponding TE biopsy) were analysed in parallel with clinical PGT-A. In this case, results of cfeDNA analysis from SEM + BF were concordant with the TE biopsy findings. Since that first birth, there are more recent reports on pregnancy outcomes where, either all three sources of DNA (removal BF using a microinjection pipette, sampling of SEM and TE biopsy in parallel) were analyzed in a clinical setting on January 29th, 2018^26^ or two sources of DNA (SEM and TE biopsy in parallel) were analysed, reported in 2019^34^.

In summary, our results suggest that miPGT-A, utilizing combined blastocoel fluid and embryo culture medium has the potential to be superior to TE biopsy for PGT-A for routine clinical use in the future.

## Materials and Methods

### Ethics approval

This research received approval from the University of Toronto Research Ethics Board (IRB #30251). Informed consent was obtained for all patients included in this study. All experiments were performed in accordance with the relevant guidelines and regulations.

### Patients and samples

**Figure 3:**
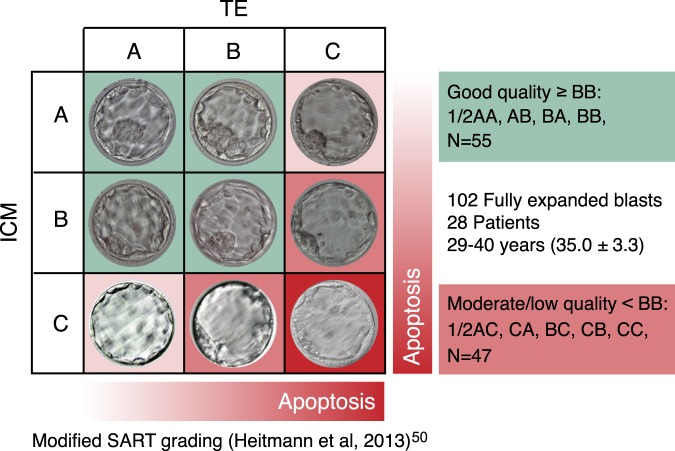
Simplified morphological scoring of blastocyst categorized them into two groups: good quality (≥BB) and moderate/low quality (<BB). The rate of apoptotic events is higher in lower morphologic grade embryos.

Combined spent embryo culture medium and blastocoel fluid samples (miPGT) from a total of 145 fresh blastocysts and their corresponding trophectoderm (TE) biopsy samples were analyzed for this report. These samples were from 28 patients, aged 33 to 42 years (mean 36.8 +/−3.0 years) undergoing PGT-A cycles from October 2018 to January 2019 at the CReATe Fertility Centre, Toronto, Canada. Of these, 102 miPGT samples and their corresponding TE biopsy samples were used to assess the impact of static embryo morphology on efficacy and accuracy of miPGT-A. Morphology of these blastocysts was evaluated based on the SART scoring system^50^ with small modifications. In this system, grade 1 are fully expanded and grade 2 are expanding blastocysts. Good quality embryos are considered ≥BB (i.e. AA, AB, BA, or BB) (n = 55), and moderate/low quality are <BB (i.e. AC, CA, BC, CB, or CC) (n = 47) (Fig. 3).

To evaluate the reliability, efficacy and accuracy of the SurePlex (Illumina, Vitrolife, CA, USA) whole genome amplification (WGA) system and WGA with vs. without a cell-lysis step, two aliquots of combined spent embryo culture medium and blastocoel fluid and corresponding TE biopsies from the remaining 43 blastocysts were included.

### Embryo culture

After collection of all cumulus*–*oocyte complexes, the cumulus and corona radiata cells were removed by a combination of enzymatic and mechanical (pipetting) procedures. Mature metaphase II oocytes were fertilized by intracytoplasmic sperm injection (ICSI). Following ICSI, each oocyte was placed in a culture dish containing 25 µl Sage1-Step medium with serum protein supplement (Origio, Denmark) under oil and then placed into the incubator (K Systems G210, Cooper Surgical, USA). Laser zona opening was performed on Day 4 to facilitate passage of embryonic cfeDNA into the culture media. Each laser zona-opened embryo was transferred on day 4to fresh 20 µlSage1-Step medium with serum protein supplement (Origio, Denmark) and cultured until blastocyst formation.

### Sample collection

#### Collection of spent embryo culture media and blastocoel fluid

Our minimally invasive and invasive preimplantation genetic testing (miPGT-A and PGT-A) workflow has been described previously^25,51^. In brief, when blastocyst full expansion was observed, the blastocysts were collapsed by a single laser pulse at the junction of TE cells (infrared Zilos-tk or Lykoslaser, Hamilton Thorne Biosciences, Beverley, MA) allowing release of blastocoel fluid (BF) into the media. After transferring the embryo to a biopsy dish, collection of the mixture of leaked BF together with embryo culture media (~5 µL) as one miPGT sample was done using sterile single use pipettes in sterile RNase–DNase-free PCR tubes and stored at −80 °C until analyzed. Control blank media samples were cultured under the same conditions and served as negative controls.

#### Trophectoderm cells (TE)

A corresponding TE biopsy sample from each embryo was obtained using our standard protocol^25,51^. All blastocysts were transferred to a biopsy dish containing 20 µL media under oil for biopsy. Four to six trophectoderm cells were biopsied from each blastocyst. The biopsied cells were placed immediately in RNase–DNase-free PCR tubes and stored at-80 °C until analyzed. Control blank media samples were collected as negative controls.

### Whole genome amplification, sequencing, and analysis

Whole genome amplification (WGA) was performed, according to manufacturer’s instructions, using the SurePlex WGA (VeriSeq PGS Kit, Illumina). The WGA starts with enzymatic lysis of biopsied cells or miPGT samples (5 ul SEM + BF) to release gDNA followed by a pre-amplification and amplification steps using degenerative primers for uniform random whole genome amplification.

When a cell lysis step was not performed, WGA SurePlex protocol starts with direct pre-amplification of 10ul miPGT (SEM + BF) sample. WGA products (SurePlex kit, Illumina) were quantified with the Qubit3.0-Fluorometerand their size distribution was assessed using 2100 BioAnalyzer (DNA high sensitivity chip, Agilent).

All samples were diluted to 0.2 ng/ul and a total of 1 ng from each sample and amplified using random primers. The kit contains 24 unique indexes added by amplification. Indexed DNA libraries were cleaned-up (AMPure XP beads 1:1 ratio) and normalized using magnetic beads. The normalized libraries were pooled, denatured, and sequenced using a MiSeq (single-end, 1 × 36 bp). Alignment and demultiplexing are done as part of the VeriSeq PGS protocol on MiSeq and CNV analysis and visualization were done using BlueFuse Multi (Illumina) software. Reporting was done using Hg39 reference with threshold for mosaicism of >30% and CNV changes >10 Mb.

### Statistical analysis

In our study, we analyzed the concordance rate for whole chromosome copy number abnormalities between miPGT samples and corresponding TE biopsy PGT-A samples. Results were statistically evaluated using Chi-squared and Fisher’s exact testing, with significance at p < 0.05.

## Supplementary information

Supplementary Data.

Supplementary Information.

## Data Availability

All data generated or analyzed during this study are included in this published article (and its Supplementary Information files).

## References

[1] Dahdouh EM, Balayla J, Garcia-Velasco JA (2015). Impact of blastocyst biopsy and comprehensive chromosome screening technology on preimplantation genetic screening: a systematic review of randomized controlled trials. Reproductive biomedicine online.

[2] Munne S (2018). Status of preimplantation genetic testing and embryo selection. Reproductive biomedicine online.

[3] Friedenthal J (2018). Next generation sequencing for preimplantation genetic screening improves pregnancy outcomes compared with array comparative genomic hybridization in single thawed euploid embryo transfer cycles. Fertility and sterility.

[4] Rubio, C. *et al*. Clinical application of embryo aneuploidy testing by NGS. *Biology of reproduction*, 10.1093/biolre/ioz019 (2019). 10.1093/biolre/ioz019 30721942

[5] Capalbo A (2018). Diagnostic efficacy of blastocoel fluid and spent media as sources of DNA for preimplantation genetic testing in standard clinical conditions. Fertility and sterility.

[6] Fang R (2019). Chromosome screening using culture medium of embryos fertilised *in vitro*: a pilot clinical study. Journal of translational medicine.

[7] Bredbacka, P. In ART Newsletter Vol. 6, (https://www.ovumia.fi/wp-content/uploads/2018/09/ARTNewsletter-2018_Peter_Bredbacka.pdf (2018). 2018).

[8] Guzman L (2019). The number of biopsied trophectoderm cells may affect pregnancy outcomes. Journal of assisted reproduction and genetics.

[9] Zhang S (2016). Number of biopsied trophectoderm cells is likely to affect the implantation potential of blastocysts with poor trophectoderm quality. Fertility and sterility.

[10] Maxwell SM (2016). Why do euploid embryos miscarry? A case-control study comparing the rate of aneuploidy within presumed euploid embryos that resulted in miscarriage or live birth using next-generation sequencing. Fertility and sterility.

[11] Popovic M (2018). Chromosomal mosaicism in human blastocysts: the ultimate challenge of preimplantation genetic testing?. Human reproduction (Oxford, England).

[12] Victor AR (2019). Assessment of aneuploidy concordance between clinical trophectoderm biopsy and blastocyst. Human reproduction (Oxford, England).

[13] He H (2019). Neonatal outcomes of live births after blastocyst biopsy in preimplantation genetic testing cycles: a follow-up of 1,721 children. Fertility and sterility.

[14] Sunkara SK, Antonisamy B, Selliah HY, Kamath MS (2017). Pre-term birth and low birth weight following preimplantation genetic diagnosis: analysis of 88 010 singleton live births following PGD and IVF cycles. Human reproduction (Oxford, England).

[15] Palini S (2013). Genomic DNA in human blastocoele fluid. Reproductive biomedicine online.

[16] Tobler KJ (2015). Blastocoel fluid from differentiated blastocysts harbors embryonic genomic material capable of a whole-genome deoxyribonucleic acid amplification and comprehensive chromosome microarray analysis. Fertility and sterility.

[17] Zhang Y (2016). Molecular analysis of DNA in blastocoele fluid using next-generation sequencing. Journal of assisted reproduction and genetics.

[18] Galluzzi L (2015). Extracellular embryo genomic DNA and its potential for genotyping applications. Future science OA.

[19] Wu H (2015). Medium-based noninvasive preimplantation genetic diagnosis for human alpha-thalassemias-SEA. Medicine.

[20] Stigliani S, Anserini P, Venturini PL, Scaruffi P (2013). Mitochondrial DNA content in embryo culture medium is significantly associated with human embryo fragmentation. Human reproduction (Oxford, England).

[21] Neal SA (2017). High relative deoxyribonucleic acid content of trophectoderm biopsy adversely affects pregnancy outcomes. Fertility and sterility.

[22] Shamonki MI, Jin H, Haimowitz Z, Liu L (2016). Proof of concept: preimplantation genetic screening without embryo biopsy through analysis of cell-free DNA in spent embryo culture media. Fertility and sterility.

[23] Feichtinger M (2017). Non-invasive preimplantation genetic screening using array comparative genomic hybridization on spent culture media: a proof-of-concept pilot study. Reproductive biomedicine online.

[24] Huang L (2019). Noninvasive preimplantation genetic testing for aneuploidy in spent medium may be more reliable than trophectoderm biopsy. Proceedings of the National Academy of Sciences of the United States of America.

[25] Kuznyetsov V (2018). Evaluation of a novel non-invasive preimplantation genetic screening approach. Plos one.

[26] Ben-Nagi J (2019). The First ongoing Pregnancy Following Comprehensive Aneuploidy Assessment Using a Combined Blastocenetesis, Cell Free DNA and Trophectoderm Biopsy Strategy. Journal of reproduction & infertility.

[27] Handyside AH (2016). Noninvasive preimplantation genetic testing: dream or reality?. Fertility and sterility.

[28] Zhang, J. *et al*. Less-invasive chromosome screening of embryos and embryo assessment by genetic studies of DNA in embryo culture medium. *Journal of assisted reproduction and genetics*, 10.1007/s10815-019-01603-w (2019). 10.1007/s10815-019-01603-w PMC6911138 31728811

[29] Leaver, M. & Wells, D. Non-invasive preimplantation genetic testing (niPGT): the next revolution in reproductive genetics? *Human reproduction update*, 10.1093/humupd/dmz033 (2019). 10.1093/humupd/dmz033 31774124

[30] Magli MC (2016). Preimplantation genetic testing: polar bodies, blastomeres, trophectoderm cells, or blastocoelic fluid?. Fertility and sterility.

[31] Ho JR (2018). Pushing the limits of detection: investigation of cell-free DNA for aneuploidy screening in embryos. Fertility and sterility.

[32] Jiao J (2019). Minimally invasive preimplantation genetic testing using blastocyst culture medium. Human reproduction (Oxford, England).

[33] Vera-Rodriguez M (2018). Origin and composition of cell-free DNA in spent medium from human embryo culture during preimplantation development. Human reproduction (Oxford, England).

[34] Rubio C (2019). Embryonic cell-free DNA versus trophectoderm biopsy for aneuploidy testing: concordance rate and clinical implications. Fertility and sterility.

[35] Li P (2018). Preimplantation Genetic Screening with Spent Culture Medium/Blastocoel Fluid for *in Vitro* Fertilization. Scientific reports.

[36] Belandres D, Shamonki M, Arrach N (2019). Current status of spent embryo media research for preimplantation genetic testing. Journal of assisted reproduction and genetics.

[37] Poli M (2019). Past, Present, and Future Strategies for Enhanced Assessment of Embryo’s Genome and Reproductive Competence in Women of Advanced Reproductive Age. Frontiers in endocrinology.

[38] Xu J (2016). Noninvasive chromosome screening of human embryos by genome sequencing of embryo culture medium for *in vitro* fertilization. Proceedings of the National Academy of Sciences of the United States of America.

[39] Madjunkova S *et al*. In *American Society of Human Genetics 68th Annual Meeting* 1270 (American Journal of Human Genetics, Orlando, Florida, 2018).

[40] Hammond ER, Shelling AN, Cree LM (2016). Nuclear and mitochondrial DNA in blastocoele fluid and embryo culture medium: evidence and potential clinical use. Human reproduction (Oxford, England).

[41] Dillon LW (2015). Production of Extrachromosomal MicroDNAs Is Linked to Mismatch Repair Pathways and Transcriptional Activity. Cell reports.

[42] Galan A (2010). Functional genomics of 5- to 8-cell stage human embryos by blastomere single-cell cDNA analysis. Plos one.

[43] Battaglia R (2019). Identification of extracellular vesicles and characterization of miRNA expression profiles in human blastocoel fluid. Scientific reports.

[44] Rule K, Chosed RJ, Arthur Chang T, David Wininger J, Roudebush WE (2018). Relationship between blastocoel cell-free DNA and day-5 blastocyst morphology. Journal of assisted reproduction and genetics.

[45] van Niel G, D’Angelo G, Raposo G (2018). Shedding light on the cell biology of extracellular vesicles. Nature reviews. Molecular cell biology.

[46] Vyas P, Balakier H, Librach CL (2019). Ultrastructural identification of CD9 positive extracellular vesicles released from human embryos and transported through the zona pellucida. Systems biology in reproductive medicine.

[47] Zeng M, Su S, Li L (2018). The effect of laser-assisted hatching on pregnancy outcomes of cryopreserved-thawed embryo transfer: a meta-analysis of randomized controlled trials. Lasers in medical science.

[48] Darwish E, Magdi Y (2016). Artificial shrinkage of blastocoel using a laser pulse prior to vitrification improves clinical outcome. Journal of assisted reproduction and genetics.

[49] Mukaida T, Oka C, Goto T, Takahashi K (2006). Artificial shrinkage of blastocoeles using either a micro-needle or a laser pulse prior to the cooling steps of vitrification improves survival rate and pregnancy outcome of vitrified human blastocysts. Human reproduction (Oxford, England).

[50] Heitmann RJ, Hill MJ, Richter KS, DeCherney AH, Widra EA (2013). The simplified SART embryo scoring system is highly correlated to implantation and live birth in single blastocyst transfers. Journal of assisted reproduction and genetics.

[51] Fuchs Weizman N (2019). Towards Improving Embryo Prioritization: Parallel Next Generation Sequencing of DNA and RNA from a Single Trophectoderm Biopsy. Scientific reports.

