# Supplementary material for: Minimally Invasive Cell-Free Human Embryo Aneuploidy Testing (miPGT-A) Utilizing Combined Spent Embryo Culture Medium and Blastocoel Fluid –Towards Development of a Clinical Assay
Source: Sci Rep. 2020 Apr 29;10:7244. doi: 10.1038/s41598-020-64335-3 (PMC7190856; doi:10.1038/s41598-020-64335-3)
Supplement: Supplementary file 2 — Supplementary Information. [file 41598_2020_64335_MOESM2_ESM.pdf]

MINIMALLY INVASIVE CELL-FREE HUMAN EMBRYO ANEUPLOIDY TESTING (miPGT-A) UTILIZING COMBINED SPENT EMBRYO CULTURE MEDIUM AND BLASTOCOEL FLUID –TOWARDS DEVELOPMENT OF A CLINICAL ASSAY

Valeriy Kuznyetsov[1*#], Svetlana Madjunkova[1*#], Rina Abramov[1], Ran Antes[1], Zenon Ibarrientos[1], Gelareh Motamedi[1], Afsaneh Zaman[1], Iryna Kuznyetsova[1]& Clifford L. Librach[1,2,3,4]

[1]CReATe Fertility Centre, Toronto, Canada. [2]Department of Obstetrics and Gynecology, University of Toronto, Toronto, ON, Canada. [3]Department of Physiology and Institute of Medical Sciences, University of Toronto, Toronto, ON, Canada. [4]Department of Gynecology, Women's College Hospital, Toronto, ON, Canada.
*Valeriy Kuznyetsov and Svetlana Madjunkova contributed equally
#Correspondence and requests for materials should be addressed to V.K. (email: valeriy@createivf.com) or S.M. (email: svetlana@createivf.com)

#Svetlana Madjunkova MD, PhD
Director, Reproductive Genetics
CReATe Fertility Centre
790 Bay Street, suite 420
M5G1N8, Toronto
Canada
Phone: +1-416-323-7727
Email: svetlana@createivf.com

Supplementary Table S1. Summary of NGS results from chromosomal copy number analysis from miPGT-1 (WGA with cell lysis), miPGT-2 (WGA without cell lysis) and TE biopsy samples obtained from the same blastocyst

Embryo number	TE biopsy	miPGT-1	WGA-DNA (ng/µl)*	miPGT-2	WGA-DNA (ng/µl)*
1	XX; -13	XX; -13	32.3	XX; -13	36.2
2	XY; +11, mosaic: +10q23.31-q26.3 (44.3Mb, 50%)	XY; +10, +11, +16	52.4	XY; +10, +11, +16	19.5
3	XX; normal	XX; normal	85.9	XX; normal	28.7
4	XX; normal	XX; normal	32.9	*Inconclusive*	47.8
5	XY; -19	XY; -19	36.2	XY; -19	43.9
6	XX; -3p26.3-p22.1 (39.8Mb)	*Inconclusive*	45.0	*Inconclusive*	13.2
7	XX; mosaic -8 (40%)	XX; normal	47.3	XX; normal	34.4
8	XY; -17, +21, mosaic: +1p31.1-p.21.1 (39Mb, 30%)	XY; -17, +21	29.2	XY; -17, +21	40.0
9	XY; normal	XY; normal	41.4	XY; normal	28.2
10	XY; normal	XY; normal	31.4	XY; normal	14.1
11	XX; normal	*Chaotic DNA signal*	19.7	*Chaotic DNA signal*	10.2
12	XY; mosaic: -16 (60%)	XY; mosaic: -16 (70%)	32.9	XY; mosaic: -16 (50%)	14.7
13	XY; mosaic: -1 (40%), -6 (40%)	XY; normal	31.5	XY; normal	29.2
14	XY; +16	XY; +16	28.8	XY; +16	46.1
15	XX; normal	XX; normal	18.1	XX; normal	12.8
16	XX; normal	XX; normal	34.1	XX; normal	36.9
17	XX; normal	XX; normal	42.9	XX; normal	41.2
18	XY; -16	XY; -16	73.1	XY; -16	67.7
19	XY; normal	XY; normal	48.3	XY; normal	38.0
20	XX; normal	*Inconclusive*	40.7	*Inconclusive*	22.2
21	XX; normal	XX; mosaic segmental: +1q21.2-q32.1	69.7	XX; mosaic segmental: +1q21.2-q32.1	60.4
22	XY; normal	XY; normal	34.1	XY; normal	17.2
23	*Chaotic DNA signal*	XY; +22	33.9	XY; +22	32.9
24	XY; normal	XY; normal	79.1	XY; normal	60.5
25	XY; normal	XY; normal	67.2	XY; normal	45.8
26	XY; +19	XY; +19	80.1	XY; +19	72.7
27	XX; +22	XX; +22	51.9	XX; +22	34.2
28	XX; normal	XX; normal	18.5	XX; normal	14.7
29	XX; -19; mosaic -16 (40%), -18 (20%)	XX; +18; mosaic -16 (70%), -19 (60%)	39.5	XX; +18; mosaic -16 (70%), -19 (60%)	31.3
30	XY; normal	XY; normal	15.7	XY; normal	15.7
31	XY; +21	XY; +21	16.6	XY; +21	29.9
32	XY; normal	XY; normal	37.7	XY; normal	39.5
33	*Chaotic DNA signal*	XY; normal	17.7	XY; normal	18.4
34	XX; normal	XX; normal	31.3	XX; normal	30.9
35	XY; normal	XY; normal	16.1	XY; normal	17.2
36	XY; -22	XY; -22	17.7	XY; -22	18.5
37	XY; -8q; Mosaic (-8p, 35%)	XY; -8q; +8p	45.8	XY; -8q; +8p	38.6
38	XY; mosaic -22 (30%)	XY; mosaic -22 (30%)	23.9	XY; normal	19.4
39	XX; +22	XX; normal	31.2	XX; normal	22.8
40	XX; +13	XX; -20	6.8	XX; -20	12.1
41	XX; +13	XX; +13	6.3	*Inconclusive*	33.1
42	XY; normal	XY; normal	30.5	XY; normal	61.4
43	XX; +9**	XX; -9**	28.4	XX; -9**	56.4

*Concentrated WGA-DNA

**Aneuploid-complementary in term of gain versus loss of chromosome 9 between TE biopsy and both miPGT samples
